# Identification and resolution of drug-related problems among diabetic patients attending a referral hospital: a prospective observational study

**DOI:** 10.1186/s40545-021-00332-9

**Published:** 2021-06-11

**Authors:** Tadesse Sheleme, Tamiru Sahilu, Desalegn Feyissa

**Affiliations:** 1 Department of Pharmacy, College of Health Science, Mettu University, Mettu, Ethiopia; 2 Department of Pharmacy, College of Health Science, Assosa University, Assosa, Ethiopia; 3 Department of Clinical Pharmacy, School of Pharmacy, College of Medicine and Health Science, Mizan-Tepi University, Mizan-Aman, Ethiopia

**Keywords:** Diabetes, Drug-related problems, Ethiopia

## Abstract

**Background:**

People living with diabetes are more vulnerable to drug-related problems due to the presence of multiple diseases. This study aimed to identify drug-related problems and contributing factors among diabetic patients.

**Methods:**

This study used a prospective observational study design. The study was conducted among diabetic patients during follow-up at Mettu Karl Referral Hospital from 15 April to 09 August 2019. The consecutive sampling was utilized to collect data. The identification of drug-related problems was performed using the Pharmaceutical Care Network Europe version 8.03. Following data collection, data were entered into Epidata manager version 4.4.2 and exported to the SPSS version 24.0 for analysis. Multivariable logistic regression analysis was done to identify predictors of drug-related problems.

**Results:**

A total of 330 people with diabetes were included in the study, among whom 279 (84.5%) had at least one drug-related problem. A total of 455 drug-related problems were identified. Effects of drug treatment not being optimal (52.7%) and untreated symptoms or indications (30.1%) were the most commonly identified drug-related problems. About 865 interventions were provided for identified drug-related problems and 79.8% was accepted. Diabetes duration $\ge 7$ years [AOR = 2.02; 95% CI (1.06, 3.85); *p* = 0.033] and the presence of comorbidity [AOR: 2.33; 95% CI (1.18, 4.60); *p* = 0.015] were factors identified as predictors of drug-related problems.

**Conclusion:**

The present study identified that drug-related problems are common among diabetic patients. Effects of drug treatment not being optimal and untreated symptoms or indications were the most commonly identified drug-related problems. Longer diabetes duration and the presence of comorbidities were predictors of drug-related problems.

## Background

Diabetes is a metabolic disorder characterized by hyperglycemia due to defects in insulin secretion and/or action [1]. Its prevalence has been steadily increasing throughout the world [2]. In 2019, the International Diabetes Federation (IDF) estimated 463 million adult people with diabetes worldwide. The IDF also estimated 19.4 million adults living with diabetes in Africa [3]. In Ethiopia, there are large numbers of people living with diabetes with an estimated 2.6 million [4]. Diabetes is a global health problem and an economic burden worldwide [2]. Diabetes-related global healthcare expenditure was 850 billion USD in 2017. Globally, diabetes contributed about 5 million deaths among the adult population in 2017. Similarly, it resulted 6% of all-cause mortality in the Africa region [5].

Although pharmacotherapy plays a major role in the cure, prevent, or diagnose diseases, it can expose patients to drug-related problems (DRPs) [6]. According to the Pharmaceutical Care Network Europe (PCNE) classification of DRPs volume 8.03, DRP is defined as “an event or circumstance involving drug therapy that actually or potentially interferes with desired health outcomes” and it classifies them into three primary domains, including treatment effectiveness problem, treatment safety and others [7]. Drug therapy problems are a consequence of a patient’s drug-related needs that have gone unmet. They are central to pharmaceutical care practice [8].

Different studies were conducted to identify drug therapy problems among people living with diabetes. For example; a study done in Jordan identified that 81.2% of study participants had at least one DRP [9]. A study from Malaysia indicated that 91.8% of diabetic patients had at least one DRP [10]. Averaging 2.1 drug therapy problems per patient were identified by Ogbonna et al. in Nigeria [11]. In Ethiopia, some studies were conducted to identify DRPs among diabetic patients. Accordingly, a study conducted in Addis Ababa showed that 45.9% of participants experienced drug therapy problems [12]. The presence of DRPs in Wolaita Soddo and Jimma was 83.1% and 88%, respectively [13, 14].

The consequences of DRPs can be increased hospitalizations, emergency department visits, additional physician office visits, and additional prescriptions [6]. DRPs interfere with patient optimal therapeutic outcomes and may be associated with higher morbidity, mortality, and healthcare cost [15]. It is identified that cost-related morbidity and mortality due to drug therapy problems exceeds the cost of the medications themselves [16]. A study showed that the economic burden due to drug-related morbidity and mortality in the United States (U.S) was $177.4 billion annually [6].

Several factors could contribute to drug therapy problems occurrence. Diabetes, its complications, and comorbid conditions cause patients to require multiple drug therapy. This results in diabetic patients more vulnerable to drug-related problems [1, 2]. Multiple drug therapy has been identified as a risk of occurrence of drug therapy problems [17]. A study revealed that liver or renal dysfunction can cause drug therapy problems through the alteration of the pharmacokinetics of diabetic medications [10]. It is also observed that older age, the presence of comorbidities, polypharmacy, and history of hospitalization are significantly associated with the occurrence of drug therapy problems [13]. DRPs can occur at any stage of medication use processes. However, lack of proper follow-up and reassessment of medical treatment by the physician is also a major problem [6].

The purpose of identifying DRPs is to help patients achieve their goals of therapy and realize the best possible outcomes from drug therapy. If not resolved, drug therapy problems have clinical consequences [8]. Clinical pharmacists play a crucial role in healthcare settings by identifying and resolving DRPs. The active role of the clinical pharmacists in healthcare settings has promoted improvement in medication use, thus maximizing the desired clinical outcomes while avoiding the unwanted effects of medication therapy with reduced cost [18]. Clinical pharmacists can effectively identify and prevent clinically significant DRPs [19].

With the advances in pharmacotherapy worldwide, understanding the nature of DRPs as well as the role of clinical pharmacists in identifying, preventing, and resolving of DRP is useful in preparing interventional strategies to reduce DRPs. The studies on DRPs among diabetic patients in sub-Saharan Africa particularly in Ethiopia were mainly focused on type 2 diabetes. And also the majority of studies conducted in Ethiopia were cross-sectional studies. The present study includes both type 1 and 2 diabetes and it was a prospective observational study. Additionally, Mettu Karl referral hospital provides services for about 2.5 million people from the catchment area. Due to its location, the hospital is serving the population from three different regions of the country, unlike most of other hospitals which service mainly population from a single region. Despite such a large hospital which services huge number population, there was no study conducted to identify and resolve DRPs. Hence, this study aimed to identify DRPs and predictors among people living with diabetes.

## Materials and methods

### Study design, setting and population

Hospital-based prospective observational study was conducted from April 15 to August 09, 2019. It was conducted at the ambulatory clinic of Mettu Karl Referral Hospital. The hospital is found in Mettu town of Oromia region in Southwest Ethiopia at 600 km from Addis Ababa, the capital city of Ethiopia. It serves about 2.5 million people from different regions of the country. The hospital health service covers the outpatient department, inpatient services, critical care, and emergency intervention unit. It provides health services for approximately 13,453 inpatient and 80,000 outpatient attendances a year.

The source population was all diabetic patients on follow-up at Mettu Karl Referral Hospital. The study population was all adult diabetic patients who visited Mettu Karl Referral Hospital during the data collection period and fulfilled the inclusion criteria. Type 1 and type 2 diabetic patients with age $\ge$ 18 years and who started taking antidiabetic medications were included. The 1-month follow-up schedule was used for data collection as the majority of patients revisit the hospital every 1 month. Diabetic patients who were not willing to participate in the study and who were not fasting were excluded.

This work was done alongside our recently published paper on glycemic control by Sheleme et al. [20]. Primitively, the study had glycemic control and drug-related problems as primary outcomes. The sample size calculation was done considering both outcomes and glycemic control provided us the maximum sample size. Thus, the proportion of poor glycemic control (59.4%) which reported from a study conducted in Jimma University Medical Center was used. The other parameters were 95% confidence interval (CI), 5% margin of error, and 10% nonresponse rate. The total number of diabetic patients with follow-up at Mettu Karl Referral Hospital was 1560. The required sample size was estimated using the single population formula and 330 was obtained after considering the correction formula. A consecutive sampling technique was used to collect data from patients who fulfilled the inclusion criteria.

### Study variables

The dependent variable was the presence of drug-related problems. The independent variables included socio-demographic variables (age, sex, education status, occupation, and residence), family history diabetes, duration of diabetes, comorbidities, diabetes-related complications, glycemic control status, and type of medications used.

### Data collection tool and procedure

The data collection was performed using a structured questionnaire and abstraction format. The questionnaire and data extraction format included patient details, investigations, medications, and clinical characteristics. The data abstraction format was used to collect the current medications a patient was taking, and disease-related data. The questionnaire was translated into Afan Oromo and Amharic languages to interview the patients. Data were collected by one nurse, one pharmacist and two clinical pharmacists. The two formers (nurse and pharmacist) interviewed the patients to get their socio-demographic characteristics and collected necessary data from patients’ medical records. The clinical pharmacists evaluated DRPs and forwarded recommendations for attending physician in order to resolve identified DRPs. Another two clinical pharmacists supervised the study alongside principal investigator to ensure the quality of data collection.

Fasting blood sugar (FBS) was measured at baseline, month-1, month-2 and month-3 of the study period. The baseline FBS was obtained on the 1st day of the patient visited hospital during the study period. The month-1 FBS was taken on the next month of the initial visit in the study period. The month-2 and month-3 FBS were obtained on the 2nd and 3rd month of the initial visit of the study period, respectively. The average of FBS measurements of 3 consecutive months was taken to categorize the diabetic patient’s blood glucose control as achieved or not achieved.

### Identification of DRPs

DRP was the primary outcome of the study. The classification of DRPs and their causes was done according to PCNE classification of DRPs volume 8.03. It classifies them into three primary domains including treatment effectiveness, treatment safety, and others (cost-effectiveness of the treatment, unnecessary drug treatment and unclear problem/complaint [7]. The identification of DRPs was performed by independent clinical pharmacists. The DRPs were assessed at baseline and then every month for 3 months of the study period. For identified DRPs, clinical pharmacists provided recommendations to a physician for adjustment, suggested a need to further investigate a patient’s condition, gave counseling to patients, and caregivers and encouraged the patients for drug adherence.

### Data quality assurance

The questionnaire was pretested on 17 diabetics at the ambulatory clinic of Jimma University Medical Center to check its consistency, applicability, and understandability. Four data collectors and two supervisors were trained for 2 days. Unclear and misunderstood questions were modified before data collection. All completed data collection forms were checked for their completeness, consistency, clarity, and accuracy by the principal investigator on daily based.

### Data processing and statistical analysis

Data were first coded and edited properly by the principal investigator. Then, the data were entered into Epidata Manager version 4.4.2 and double entry verification was made. Data were exported to Statistical Package for Social Science (SPSS) version 24.0 for analysis. A multivariable logistic regression model was done to identify predictors of DRPs. The variables were considered as predictors if statistically significant at *p*-value < 0.05.

### Definition of terms

DRP is an event involving drug therapy that actually or potentially interferes with desired health outcomes [7].

The clinical pharmacists’ intervention outcomes were categorized using PCNE version 8.03 as the DRPs were ‘solved’, ‘partially solved’, or ‘not solved’ [7].

## Results

### Socio-demographic and clinical characteristics of the study participants

A total of 330 adult diabetic patients were included in this study. One hundred ninety-eight (60.0%) participants were males. The age of 156 (47.3%) study participants was found within the range of 41 to 60 years. The educational status of the study population showed that 114 (34.5%) had attained primary school education. In terms of occupation, 113 (34.2%) study participants were farmers. More than half (53.6%) of the respondents were urban residents. The mean ± SD diabetes duration of participants was 7.72 ± 5.91 years and 47.6% had a duration of 7 or more years. Comorbid diseases and diabetes complications were identified in 43.3% and 38.5%, respectively. On assessing the glycemic control status, 72.7% of participants did not achieve the recommended goals of glycemic control (Table 1).

**Table 1:** Socio-demographic and clinical characteristics of participants on follow-up at Mettu Karl Referral Hospital, Southwest Ethiopia, 2019

Variables	Categories	Type 1 DM	Type 2 DM	Total *n* (%)
		*n* (%)	*n* (%)	
Sex	Male	87 (43.9)	111 (56.1)	198 (60.0)
	Female	41 (31.1)	91 (68.9)	132 (40.0)
Age (years)	18–40	70 (72.9)	26 (27.1)	96 (29.1)
	41–60	45 (28.8)	111 (71.2)	156 (47.3)
	> 60	13 (16.7)	65 (83.3)	78 (23.6)
Educational status	No formal education	31 (43.7)	40 (56.3)	71 (21.5)
	Primary education	47 (41.2)	67 (58.8)	114 (34.5)
	Secondary education	32 (49.2)	33 (50.8)	65 (19.7)
	Tertiary education	18 (22.5)	62 (77.5)	80 (24.2)
Occupation	Farmers	55 (48.7)	58 (51.3)	113 (34.2)
	Merchants	43 (44.3)	54 (55.7)	97 (29.4)
	Employees	12 (26.7)	33 (73.3)	45 (13.6)
	House wives	6 (14.6)	35 (85.4)	41 (12.4)
	Retired	5 (20.0)	20 (80.0)	25 (7.6)
	Others^a^	7 (77.8)	2 (22.2)	9 (2.7)
Residence	Urban	55 (31.1)	122 (68.9)	177 (53.6)
	Rural	73 (47.7)	80 (52.3)	153 (46.4)
Family history of DM	Yes	20 (21.1)	75 (78.9)	95 (28.8)
	No	108 (46.0)	127 (54.0)	235 (71.2)
Duration of DM (years)	< 7	59 (17.9)	114 (34.5)	173 (52.4)
	$\ge$ 7	69 (20.9)	88 (26.7)	157 (47.6)
Presence of comorbidities	Yes	48 (33.6)	95 (66.4)	143 (43.3)
	No	80 (42.8)	107 (57.2)	187 (56.7)
Type of comorbidities	Hypertension	43 (33.3)	86 (66.7)	129 (39.1)
	Heart failure	4 (20.0)	16 (80.0)	20 (6.1)
	Asthma	4 (50.0)	4 (50.0)	8 (2.4)
	Ischemic heart disease	0 (0.0)	7 (100.0)	7 (2.1)
	Others^ b^	2(25.0)	6 (75.0)	8 (2.4)
Presence of complications	Yes	35 (27.6)	92 (72.4)	127 (38.5)
	No	93 (45.8)	110 (54.2)	203 (61.5)
Type of complications	Neuropathy	14 (17.7)	65 (82.3)	79 (23.9)
	Retinopathy	16 (39.0)	25 (61.0)	41 (12.4)
	Nephropathy	6 (18.8)	26 (81.2)	32 (9.7)
	Others^ c^	1 (16.7)	5 (83.3)	6 (1.8)
Glycemic control	Achieved (80–130 mg/dl)	24 (7.3)	66 (20.0)	90 (27.3)
	Unachieved (> 130 mg/dl)	104 (31.5)	136 (41.2)	240 (72.7)

*DM* diabetes mellitus

^a^Daily laborers, drivers and students

^b^Stroke, toxic goiter and human immunodeficiency virus (HIV)

^c^Impotency and foot ulcer

### Medication usage patterns among study population

The combination metformin and glibenclamide was prescribed to 27.6% of participants. Insulin injection (40.3%) was the most frequently used monotherapy. Oral antidiabetic medications with insulin were given in 14.2% of the study participants. Cardiovascular drugs were the most commonly co-prescribed medications, of which 26.4% were angiotensin converting enzyme inhibitors (Table 2).

**Table 2:** Medication usage patterns among participants in the study conducted at Mettu Karl Referral Hospital, 2019

Variables	Type of medications	*n*	%
Antidiabetic medications	Metformin	50	15.2
	Glibenclamide	9	2.7
	Insulin	133	40.3
	Metformin + glibenclamide	91	27.6
	Metformin + insulin	47	14.2
Cardiovascular medications	Angiotensin converting enzyme inhibitors	87	26.4
	Calcium channel blockers	32	9.7
	Diuretics	34	10.3
	Beta blockers	25	7.6
Lipid-lowering agents	Statins	40	12.1
Antiplatelet	Aspirin	33	10.0
Antidepressant	Amitriptyline	9	2.7
Bronchodilator	Salbutamol	8	2.4
Antiretroviral therapy	Tenofovir–lamivudine–efavirenz	4	1.2
Others	Propylthiouracil, spironolactone and antibiotics	6	1.8
Number of prescribed medications	< 5	308	93.3
	≥ 5	22	6.7

### Types, causes of DRPs and interventions

A total of 455 DRPs were identified. Among the 330 study participants, 279 (84.5%) had at least one DRP and the mean number of DRPs per patient was 1.38 ± 0.85. Problems regarding treatment effectiveness were the most common DRPs encountered, with the effect of drug treatment not optimal being the most frequent problem (52.7%), followed by untreated symptoms or indications which counted for 30.1% (Table 3).

**Table 3:** Drug-related problems among participants at Mettu Karl Referral Hospital, 2019

Code	Detailed classification	*n* (%)
P	Problem	455 (100)
P1	Treatment effectiveness	393 (86.4)
P1.1	No effect of drug treatment	16 (3.5)
P1.2	Effect of drug treatment not optimal	240 (52.7)
P1.3	Untreated symptoms or indication	137 (30.1)
P2	Treatment safety	42 (9.2)
P2.1	Adverse drug event (possibly) occurring	42 (9.2)
P3	Other	20 (4.4)
P3.1	Problem with cost-effectiveness of the treatment	12 (2.6)
P3.2	Unnecessary drug treatment	8 (1.8)
C	Cause	527 (100)
C1	Drug selection	170 (32.3)
C1.1	Inappropriate drug according to guidelines/formulary	12 (2.3)
C1.2	Inappropriate drug (within guidelines but otherwise contra-indicated)	4 (0.8)
C1.3	No indication for drug	8 (1.5)
C1.4	Inappropriate combination of drugs	6 (1.1)
C1.5	Inappropriate duplication of therapeutic group or active ingredient	3 (0.6)
C1.6	No or incomplete drug treatment in spite of existing indication	137 (26.0)
C2	Drug form	0 (0.0)
C3	Dose selection	80 (15.2)
C3.1	Drug dose too low	28 (5.3)
C3.2	Drug dose too high	18 (3.4)
C3.3	Dosage regimen not frequent enough	7 (1.3)
C3.4	Dosage regimen too frequent	11 (2.1)
C3.5	Dose timing instructions wrong, unclear or missing	16 (3.0)
C4	Treatment duration	15 (2.8)
C4.1	Duration of treatment too short	9 (1.7)
C4.2	Duration of treatment too long	6 (1.1)
C5	Dispensing	45 (8.5)
C5.1	Prescribed drug not available	10 (1.9)
C5.2	Necessary information not provided	35 (6.6)
C6	Drug use process	58 (11.0)
C6.1	Inappropriate timing of administration or dosing intervals	27 (5.1)
C6.2	Drug under-administered	17 (3.2)
C6.3	Drug over-administered	3 (0.6)
C6.4	Drug not administered at all	11 (2.1)
C7	Patient related	159 (30.2)
C7.1	Patient uses/takes less drug than prescribed or does not take the drug at all	25 (4.7)
C7.2	Patient uses/takes more drug than prescribed	4 (0.8)
C7.4	Patient uses unnecessary drug	3 (0.6)
C7.6	Patient stores drug inappropriately	71 (13.5)
C7.7	Inappropriate timing or dosing intervals	29 (5.5)
C7.8	Patient administers/uses the drug in a wrong way	5 (0.9)
C7.9	Patient unable to use drug/form as directed	22 (4.2)
I	Interventions	865 (100.0)
I1	At prescriber level	351 (40.6)
I1.1	Prescriber informed only	43 (5.0)
I1.2	Prescriber asked for information	22 (2.5)
I1.3	Intervention proposed to prescriber	71 (8.2)
I1.4	Intervention discussed with prescriber	215 (24.9)
I2	At patient level	204 (23.6)
I2.1	Patient (drug) counseling	161 (18.6)
I2.3	Patient referred to prescriber	32 (3.7)
I2.4	Spoken to family member/caregiver	11 (1.3)
I3	At drug level	310 (35.8)
I3.1	Drug change	25 (2.9)
I3.2	Dosage change	84 (9.7)
I3.4	Instructions for use change	43 (5.0)
I3.5	Drug pause or stop	21 (2.4)
I3.6	Drug start	137 (15.8)
A1	Intervention acceptance	690 (79.8)
A1.1	Intervention accepted and fully implemented	608 (88.1)
A1.2	Intervention accepted, partially implemented	64 (9.3)
A1.3	Intervention accepted but not implemented	18 (2.6)
A2	Intervention not accepted	175 (20.2)
A2.1	Intervention not accepted: not feasible	8 (4.6)
A2.2	Intervention not accepted: no agreement	167 (95.4)
O	Outcome of intervention	
O1.1	Problem totally solved	322 (70.8)
O2.1	Problem partially solved	44 (9.7)
O3.2	Problem not solved, lack of cooperation of prescriber	85 (18.7)
O3.4	No need or possibility to solve problem	4 (0.9)

*P* problem, *C* cause, *I* intervention, *A* acceptance; *O* outcome

There were about 527 identified causes of DRPs. The most common causes of DRPs were related to drug selection (32.3%) with no or incomplete drug treatment in spite of existing indications being the most common cause (26.0%). Patient-related problems (30.2%) were the second most common cause of DRPs (Table 3).

**Fig. 1:**
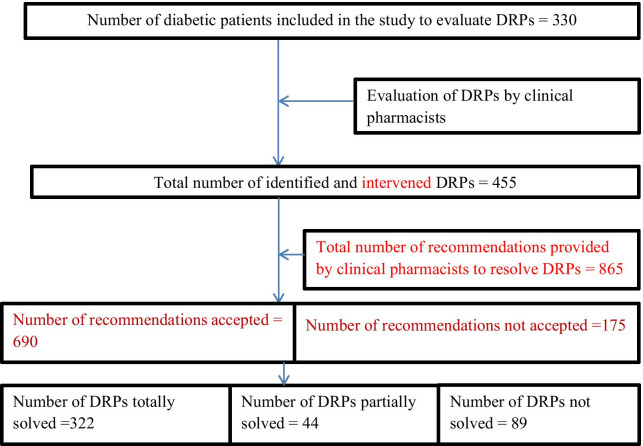
DRPs evaluation flowchart

A total of 865 interventions were provided. Most of the interventions were given at the prescriber level (40.6%), followed by at the drug level (35.8%) and at the patient (23.6%). The acceptance rate of the provided interventions was 79.8%. Among the identified DRPs, about 322 (70.8%) was fully resolved. On the other hand, 44 (9.7%) DRPs were partially resolved (Table 3, and Fig. 1).

### Predictors of DRPs among study population

The multivariable analysis indicated that longer duration of diabetes and the presence of comorbidities were predictors of DRPs. Study participants who have lived with diabetes for 7 years or more were about two times more likely to have DRPs when compared to those who have lived with diabetes for less than 7 years [AOR = 2.02; 95% CI (1.06, 3.85); *p* = 0.033]. The participants who had comorbidity were 2.3 times more likely to have DRPs [AOR: 2.33; 95% CI (1.18, 4.60); *p* = 0.015] compared to those who had no comorbidity (Table 4).

**Table 4:** Multivariable analysis results for the variables associated with DRPs

Variable	Category	DRP		AOR (95% CI)	*p*-value
		No	Yes		
Diabetes duration (years)	< 7	35	138	1	
	$\ge 7$	16	141	2.019 (1.059, 3.850)	0.033*
Comorbidity	No	13	130	1	
	Yes	38	149	2.333 (1.182, 4.604)	0.015*
Age (years)	18–40	22	74	1	
	41–60	23	133	1.385 (0.705, 2.721)	0.344
	> 60	6	72	2.381 (0.872, 6.504)	0.091
Educational status	No formal education	7	64	2.207 (0.816, 5.965)	0.119
	Primary education	14	100	2.477 (1.068, 5.746)	0.058
	Secondary education	14	51	1.319 (0.552, 3.154)	0.534
	Tertiary education	16	64	1	

*AOR* adjusted odds ratio, *DRP* drug-related problem

^*^Statically significant at *p*-value less than 0.05

## Discussion

DRPs are considered as serious, expensive, and complicate the health-care system. They are common among people living with chronic illness like diabetic patients [9]. If drug therapy problems are not addressed, they can lead to clinical complications [21]. Detecting and resolving DRPs is important to ensure that patients achieve the optimal therapeutic goals [21].

Overall, the current study showed that 84.5% of the study participants had at least one DRP. This result is similar to previously conducted studies, including a study conducted in Jordan (81.2%) and in Ethiopia (88%) [9, 14]. However, it is higher than the finding of another study previously conducted in Ethiopia, which reported 64.2% of DRPs among diabetic patients attending follow-up [22]. The discrepancy may be due to the difference in the method used to assess and classify DRPs. In our study, PCNE classification of DRPs was used, while the previous study utilized Cipolle’s method of DRPs classification system.

The present study showed that treatment effectiveness (86.4%) was the main category of DRP identified and treatment safety (9.2%) was the second most commonly encountered. The effects of drug treatment not being optimal (52.7%) and untreated symptoms or indications (30.1%) were the most frequently observed treatment effectiveness problem. Drug selection (32.3%) and dose selection (15.2%) were the main causes of DRPs. Our study findings are consistent with a previous study done in Ethiopia which showed that the effect of drug treatment not being optimal (49.2%), and untreated indication and symptoms (21.1%) were the most common type of identified DRPs [23]. It is also consistent with a study conducted in China, which reported that treatment effectiveness (53.71%) and treatment safety (33.90%) were the most common DRPs encountered. The study also showed that drug selection (71.43%) and dose selection (20.57%) were the main causes of DRPs [24].

Following the identification of DRPs, interventions were provided by the clinical pharmacists. Interventions were given at different levels including at the prescriber level, at the drug level and at the patient level. The acceptance rate of the clinical pharmacists’ recommendations was 79.8%. This is in line with a study conducted by Argaw et al. [14], which revealed that the acceptance rate of clinical pharmacist’s recommendations was 72.6%. The identification and intervention by clinical pharmacists with clinically significant DRPs, and further, the acceptance of interventions by prescribers, are evidence of the major contribution of clinical pharmacists in minimizing the occurrence of DRPs, thus implying better drug therapy for the patient [19].

In this study, multivariable analysis showed that longer diabetes duration was an independent predictor of DRPs. Study participants who have lived with diabetes for 7 years or more had more DRPs when compared to those who have lived for less than 7 years. This finding is similar with a study done in India, which reported that duration of diabetes was associated with DRPs [6]. This may be because patients with longer diabetes duration are at higher risk of developing diabetes complications, and likely to have comorbid conditions which contribute to multiple drug therapy which in turn increases the chance of drug–drug interactions, adverse drug events and nonadherence to drugs.

This study observed that the presence of comorbidity was another independent predictor of DRPs. It is similar with previous studies conducted in Ethiopia [13, 16]. A study conducted by Amit Sharma et al. [6] also reported that the presence of comorbidities were significantly associated with DRPs. In the presence of multiple medical conditions, medications are required to be initiated for those medical conditions causing the prescription of multiple drugs. The multiple drugs utilization can cause drug–drug interaction and a complex drug schedule. The frequent daily medication use and increased pill numbers may contribute to drug therapy problems.

### Strength and limitation of the study

The strength of this study was that DRPs were identified prospectively using the standardized tool PCNE version 8.03. One of the limitations of the present study was that the severity of DRPs was not determined. The second limitation was the use of FBS to assess glycemic control level since the HbA1c test was not available in the study area. The third limitation of the study was using of consecutive sampling technique which might weaken the generalization of the findings. Another limitation of the study was that some data were obtained from the patients’ medical record which might affect the quality of data.

## Conclusion

The present study identified that DRPs are common among diabetic patients attending follow-up. Patients with longer duration of diabetes and comorbidities had a higher chance of developing DRPs. Effect of drug treatment not being optimal and untreated symptoms or indications were the most commonly identified DRPs. Most of the recommendations suggested by clinical pharmacists to solve DRPs were accepted. Clinical pharmacists play a major role in identifying and resolving DRPs, and therefore it is important to strengthen clinical pharmacists’ services in health-care system.

## Data Availability

The datasets used and/or analyzed during the current study are available from the corresponding author on reasonable request.

## Abbreviations

DM: Diabetes mellitus
DRPs: Drug-related problems
FBS: Fasting blood sugar
IDF: International Diabetes Federation
PCNE: Pharmaceutical Care Network Europe
SPSS: Statistical Package for Social Science
US: United States
